# Risk-based principles for defining and managing water security

**DOI:** 10.1098/rsta.2012.0407

**Published:** 2013-11-13

**Authors:** Jim Hall, Edoardo Borgomeo

**Affiliations:** Environmental Change Institute, University of Oxford, Oxford, UK

**Keywords:** water security, risk, hazard, vulnerability, exposure, decision

## Abstract

The concept of water security implies concern about potentially harmful states of coupled human and natural water systems. Those harmful states may be associated with water scarcity (for humans and/or the environment), floods or harmful water quality. The theories and practices of risk analysis and risk management have been developed and elaborated to deal with the uncertain occurrence of harmful events. Yet despite their widespread application in public policy, theories and practices of risk management have well-known limitations, particularly in the context of severe uncertainties and contested values. Here, we seek to explore the boundaries of applicability of risk-based principles as a means of formalizing discussion of water security. Not only do risk concepts have normative appeal, but they also provide an explicit means of addressing the variability that is intrinsic to hydrological, ecological and socio-economic systems. We illustrate the nature of these interconnections with a simulation study, which demonstrates how water resources planning could take more explicit account of epistemic uncertainties, tolerability of risk and the trade-offs in risk among different actors.

## 1. Defining water security

Water security has acquired increasing prominence as a concept and objective in global water policy, though the extent to which it differs from previous framings of global water challenges is not always clear [1]. Certainly, the original definition in terms of a broad aim in which ‘every person has access to enough safe water at an affordable cost to lead a clean, healthy and productive life, while ensuring the environment is protected and enhanced’ [2, p. 12] hardly seemed to add value to existing global water objectives, for example articulated in the Millennium Development Goals. Yet, subsequent definitions have increasingly associated water security with the management of water-related risks. UN Water [3, p. 1] incorporates protection from ‘water-related disasters’ in its definition, and Mason & Calow [4] recognize the importance of variability and risk in their pragmatic set of water security indicators. Grey & Sadoff [5, p. 1] couple ‘an acceptable level of water-related risks’ with ‘the availability of an acceptable quantity and quality of water for health, livelihoods, ecosystems and production’. In this Theme Issue, Grey *et al*. [6, p. 3] take the focus on water-related risks a step further, to simply define water security as ‘tolerable water-related risk to society’. This focus upon water risks not only seems to be congruent with the language of ‘security’, but also brings theoretic, empirical and operational substance to the term ‘water security’, in ways that will be set out in this paper.

Risk is associated with the potential for undesirable outcomes to materialize. Analysis of risks therefore deals with the nature of possible undesirable outcomes and their tendency to occur. Risk-based decision making uses evidence of risks to inform individual and societal choices about which courses of action to adopt in future. It involves weighing up risks and costs, in the broadest possible sense, for a range of different actors.

In the context of water security, the focus is upon potentially harmful outcomes associated with the aquatic environment. A given water resources system (a river basin, an estuary or a continent) will have a range of water-related states (in terms of water quantity and quantity), defined at a range of spatial and temporal scales. Associated with those states will be outcomes for human health and well-being, the economy and the natural environment. We are concerned about water-related outcomes in which needs for water services (to people and the environment) are not satisfied. These are oftentimes associated with the extremes of the hydrological spectrum (droughts and floods) and harmful water quality. Harmful outcomes are a consequence of disastrous events (the ‘water-related disasters’ of the UN Water definition [3]), but may equally well be the result of chronic conditions, for example associated with salinization, hypoxia and waterlogging. Even in the absence of harmful physical aquatic conditions, individuals and communities may risk a shortage of water or sanitation services because of deficiencies of entitlement or access.

The possibility of harmful states of the aquatic environment (droughts, floods, harmful water quality, etc.) can seldom be eliminated. The notion in UN Water's definition [3, p. 1] that ‘protection against … water-related disasters’ can be ‘ensur[ed]’ denies the random nature of these events and the unbounded potential for some extremes. Risk management involves weighing likelihoods and consequences of a range of possible outcomes and engaging in societal discussion of the tolerability of risks and the willingness to pay for risk reduction, recognizing that risks are socially constructed and that there is a range of factors that determine individuals’ perception of risk [7]. Analysis of other risks to which society is exposed will help to place water-related risks in context and to identify the appropriate scale and nature of response.

The definition of tolerability of water-related risk is bound to be shaped by cultural and economic contexts and the scale and range of other risks to which a society is exposed. The threshold of tolerability will not be a crisp one. Yet, instances abound where the likelihood and consequences of water-related risks have stimulated societal and political action, be it to cope with the chronic effects of hydrological variability on the economy of Ethiopia [8], the damaging effects of floods on the Mississippi [9,10] or the degraded aquatic environment in Europe which has led to the Water Framework Directive [11]. The rubric of water security offers the potential to both analyse and generalize these instances of risk management.

In their development of indicators of water security, Lautze & Manthrithilake [1] identify five components that they consider to be critical to the concept of water security: (i) basic needs, (ii) agricultural production, (iii) the environment, (iv) risk management, and (v) independence. The ‘risk-management’ indicator is associated specifically with ‘prevention of water-related disasters’. In this paper, we argue that risk is *the* defining attribute of water security, though risk has multiple dimensions. Thus, each of Lautze and Manthrithilake's indices can be taken as an indicator of risk (i) of not satisfying basic needs (for given proportions of time and quantiles of the population), (ii) of inadequate agricultural production owing to water-related constraints, (iii) of harmful environmental impacts, and (iv) to the reliability of water supplies from the actions of neighbouring countries. Thinking of water security as the absence of intolerable risks leads to consideration of a broad range of water-related risks and context-specific evaluation of their tolerability.

Meanwhile, Cook & Bakker [12] identify four framings of water security: water availability; human vulnerability to hazards; human needs (development-related, with an emphasis on food security); and sustainability. The first three of these can be recast in terms of tolerable risk, while the last is a broad catch-all for a range of different issues. More recently, Bakker has advocated a risk-based framing, arguing that ‘the concept of risk is deployed across the biological, social, physical, and medical sciences, and is hence compatible with an interdisciplinary approach to analysing water security’.

As we seek to demonstrate in this paper, water security provides the basis for development of general frameworks for measuring and managing water-related risks. We begin by analysing in more detail the theoretical and empirical motivations for thinking of water security in terms of risk. We go on also to identify some practical attractions of this approach. We then formalize a definition of risk, in particular focusing upon the multi-attribute nature of risk and the multiple perspectives of different actors in water resources systems. A risk-based framework is also proposed for development of indicators of water security. The risk-based definition is theoretical, so we provide an application in the context of a water resources system. The application neither deals with all dimensions of water-related risks, nor does it explore different actors’ attitudes to risk. It does however demonstrate how different sources of uncertainty, including the severe uncertainty in the outputs from climate models, can be incorporated in risk-based planning decisions. We address some of the criticisms of a risk-based approach before concluding.

## 2. Why focus upon risk?

### (a) Theoretical and empirical observations

While market values of water are typically lacking [13], theory and simulation studies yield decreasing marginal values [14–16]. Water users and managers tend to be concerned with avoiding undesirable shortages (or indeed extreme flood flows) up to a point when a satisfactory quantity of water is assured and the marginal value of water declines. In other words, water users are concerned about the risk of water-related needs not being satisfied, rather than about maximizing consumption of water resources. Even though in many countries the marginal cost of domestic water supply is at or close to zero, domestic demand for water does not increase in an unbounded way. There is a limit to the number of crops that farmers can cultivate in any given year, so again their need for water is bounded and their concern is with water scarcity threatening their objectives for agricultural production. Similarly, in relation to water quality, the focus is upon achieving acceptable water quality standards within the context of given uses and environments, rather than upon achieving the best possible water quality. The concern is with the risk of standards not being satisfied.

There are uses of water that will continue to yield benefit in a more or less unbounded way. In electricity markets where hydropower substitutes for larger electricity supplies with a higher marginal cost, hydropower generators can always sell electricity. Decision makers may well wish to maximize these beneficial outcomes, but we do not regard this as a means to achieving water security (though it may be a means to achieving energy security). Indeed, it may be a threat to water security if hydropower generation increases the water-related risks to which other people and the environment are exposed.

It seems that water users are more concerned with satisfying an acceptable level of water-related risks rather than maximizing returns. In an empirical study of 22 case studies of water resources planning, Rogers & Fiering [17] identified limited use of optimization techniques. On the contrary, drawing upon the work of Simon [18], they promote robust water management decisions that maximize ‘the probability of achieving acceptable (satisfactory) outcomes’ rather than necessarily optimizing overall performance. This is the approach to decision making that Simon described as ‘satisficing’ behaviour. Robust satisficing is promoted in more contemporary analysis of water resource management in the context of a changing climate [19–21]. Robust satisficing responds to the often severe uncertainties to which decision makers often find themselves exposed, by proposing that under such conditions decision makers should seek options that satisfy minimum performance requirements under the widest range of future conditions, rather than seeking to optimize a particular performance objective [22].

**Figure 1.**
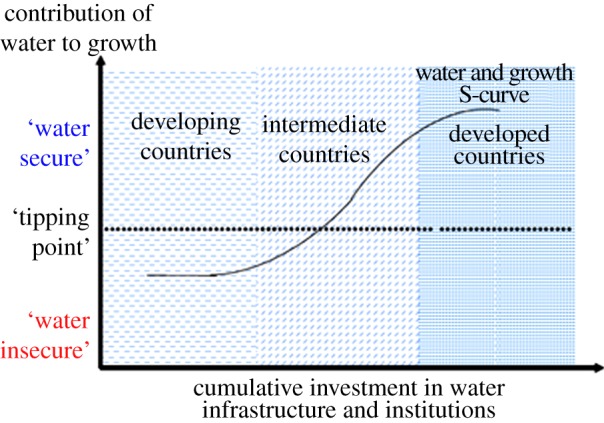
The water security S-curve [5]. (Online version in colour.)

Paralleling these arguments, at a macro-economic scale, Grey & Sadoff [5] identify a levelling off in investment in water infrastructure and institutions that accompanies a transition to water security (figure 1). In ‘water insecure’ societies, water-related risks are intolerably high and inhibit growth. As a consequence, resources for investment and institutions and infrastructure to manage the risks are constrained and the societies are ‘hostage to hydrology’. When resources are made available to manage water-related risks, the marginal benefits are high and risks are rapidly reduced. As risks go down, the marginal cost per unit of risk reduction increases, and a point is reached where the S-curve flattens off: large new investments are not justified because water-related risks are tolerably low—the country is ‘water secure’. The story does not end there however, as institutions and infrastructure can and will deteriorate if not maintained, expectations with respect to risk to people and the natural environment are dynamic and the climate is changing meaning that societies that had perceived themselves to be water secure are now facing new threats.

**Figure 2.**
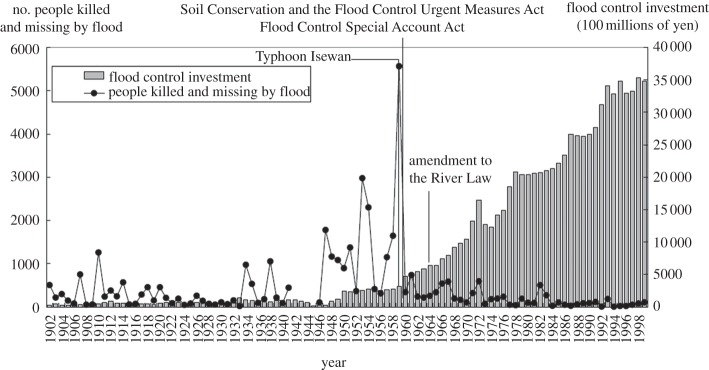
Flood damages and flood control infrastructure investment in Japan [23].

Research by the Japan Water Forum and the World Bank [23] illustrates this transition in Japan. Observe this in the plot of deaths versus flood control investment in Japan (figure 2). Flooding, caused by heavy seasonal rains as well as typhoons, had major economic impacts in Japan while the country was recovering from World War II. Flood damages occasionally exceeded 10% of gross domestic product (GDP), and in 1959 Typhoon Isewan resulted in the death of 5159 people. The risk to this rapidly growing economy was unacceptable and, from 1950 to 1975, some 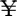
2 trillion was invested in river infrastructure (similar to the investment in railways). Since the 1970s, the impact of flood on the Japanese economy has not exceeded 1% of GDP in any year. The average annual damage is roughly 0.1% of GDP. This figure is reflected elsewhere: the IPCC's Special Report on Extreme Events [24] (SREX) reported climate-related damages from extreme events (of which hydro-meteorological risks are the largest contributor) of ‘less than 0.1% of GDP for high-income countries … during the period from 2001 to 2006’. Underlining the Grey and Sadoff S-curve, SREX reported losses of 1% of GDP for middle-income countries, which have high economic vulnerability and under-developed risk reduction, while this ratio has been about 0.3% of GDP for low-income countries which have low economic vulnerability in GDP terms because they are so poor, but high human vulnerability.

In an analysis of water-related risks through the lens of Beck's [25] *Risk Society*, Allan [26, p. 3] noted how ‘extreme natural events tend to achieve an exceptional convergence of awareness on the part of major and minor water users, as well as on the part of policy makers, legislators and of influential agents, for example the media’. Extreme events open ‘policy windows’ [27] in which policy imperatives and opportunities exist to respond to risks. This was the case in The Netherlands where the 1953 floods (in which 1836 people died) stimulated the major programme of Delta Works, which was completed in 1997. However, as we have already noted, changing societal expectations and a changing climate can stimulate re-evaluation of tolerable levels of risk, which took place in The Netherlands in the work of the Deltacommissie [28], which advocated a 10-fold increased standard of protection against flooding. As the economic case for such a high standard is at least questionable [29] and Dutch society was hardly clamouring for a reduction in risk (which is already so low as to be imperceptible to most citizens), we may speculate as to why further investment of €3bn per year was advocated. At a time when global business confidence is so weak, a bold statement of water security that The Netherlands will be open for business ‘come what may’ was perhaps more aimed at the international investor community than at domestic audiences.

The empirical cases in this exploration of the relationship between water risk and decision making have focused upon flooding (in Japan and The Netherlands). Flooding is a tractable case because the impacts can be relatively readily defined in economic terms and the costs of mitigation are largely incurred by governments so tend to be available. Grey & Sadoff [5] provide narratives of the water security transition in the context of a wider range of water-related risks, including drought risks to agriculture and salinization, with instances of countries at different stages of development. In each of these cases, we observe how choices and outcomes at a micro- and macroscale can be explained in terms of water-related risks and management response to them.

### (b) Operationalizing water security

Having advanced some theoretical and empirical motivations for thinking of water security in terms of risk, we now examine how a risk-based definition of water security can be helpful in practical terms.

Risk brings particular focus to water management problems because it is concerned with outcomes that we value. Analysis of risks provides essential evidence to enable choices between alternative courses of action. In that sense, risk is forward-looking, using analysis of risks now and in the future to inform decision making. Specifically, and crucially, risk relates the potential for a chosen action or activity (including the choice of inaction) to lead to undesirable outcomes. It thus provides the basis for deciding upon future actions, with the aim of managing water-related risks. It begs the question ‘What is it worth to reduce the risks associated with water resource systems?’ Or conversely ‘How much is an improvement in water security worth (compared to other pressing needs)?’ [4]. It does imply the need to value undesirable outcomes, which in contested settings and situations of complexity and severe uncertainty is bound to be problematic. Yet, the requirement to explore the tolerability of risks is constructive in its own right, helping to expose objectives and constraints that may not be articulated.

The forward-looking nature of risk provides the basis for promotion of proactive strategies to manage risks before disasters actually materialize. We have noted that disasters are often the stimulus for political action to reduce risk, but risk analysis can be used to provide the economic and societal rationale for reducing risk before a disaster occurs. Instances of this proactive approach are, anecdotally, rare, though that may be because they have resulted in disasters *not* occurring so are underreported. However, the Dutch Deltacommissie, mentioned above, could be taken as an instance of a proactive risk-management strategy. The Deltacommissie's work also illustrates how a risk framing can be used to think in the long term about future (uncertain) changes and plan timely responses to those changes.

A focus upon choices and outcomes distinguishes a risk-based perspective from other process-oriented approaches to water issues, notably integrated water resources management (IWRM). Proponents of IWRM [30] have argued how inclusive and flexible processes are essential to managing the multiple demands placed upon water resources. Water security complements IWRM by articulating the water-related outcomes that are of most concern to decision makers. It is argued that IWRM provides the process through which the dimensions of water security can be addressed together [31].

Definitions of risk incorporate some combination of likelihood and consequence of sets of possible outcomes as being a basis for decision making [32]. Inherent therefore is the notion that there is a range of possible outcomes (some of which may not even be foreseeable [33]) and the outcome(s) that will in practice materialize cannot be precisely forecast. This emphasis upon unpredictability (at least in deterministic terms) is particularly constructive in the context of water-related hazards, because natural variability, on time scales from minutes (e.g. for flash floods) to decades (e.g. for groundwater reserves), as well as in space, is such a distinguishing characteristic of water resources systems. Risk analysis encourages us to think about a whole range of possible future conditions, from the everyday to the extremely unlikely. Analysis of hydrological variability has long formed the basis for water resources management decisions, for example in relation to infrastructure planning and operation [34,35]. A risk-based definition of water security embeds the management of variability (and associated uncertainties) at the heart of water policy.

Traditional engineering approaches to dealing with variability have sought to identify a ‘design condition’ (for example, a reservoir storage volume or a dike crest level) and design to meet that condition [36]. A risk-based approach involves considering the full range of conditions to which a system might be exposed, including those that exceed the ‘design condition’. This places a focus upon system response to extreme conditions and the capacity to recover, i.e. resilience. Attention is drawn to the modes by which systems may fail and whether failure is catastrophic or ‘graceful’. Warning systems and emergency relief have been recognized as integral aspects of the systemic management of risks.

A risk-based perspective equally emphasizes the probability and the consequences of harmful events. It thus provides a mechanism to analyse the effectiveness of risk-management options designed to reduce the severity of the aquatic hazard and to reduce exposure and vulnerability, the two factors which determine our ability to manage or adapt to hazards. Following SREX [24, p. 3], we think of ‘vulnerability’ as the ‘propensity or predisposition to be adversely affected’, in other words the sensitivity of the system to be adversely affected by a hazard. The ‘exposure’ is the ‘presence of people; livelihoods; environmental services and resources; infrastructure; or economic, social, or cultural assets in places that could be adversely affected’. Thus measures to reduce vulnerability, through the development of coping or risk-sharing strategies (e.g. insurance), or exposure (e.g. through land use zoning) can be readily appraised within the risk-based framework. Populations that are disadvantaged in their access to water, for example owing to economic disadvantage or lack of entitlement, are regarded as being particularly vulnerable.

While risk provides a mechanism (via probabilities) for incorporating variability into management decisions, we are also concerned about processes of change, which mean that probability distributions in future may be different from the one implied by observations. Change can also influence the vulnerability and exposure to hazards, for example through socio-economic changes. A well-defined analysis of risks can be scrutinized to identify those factors that may change in future and to test the sensitivity of decisions to potential future changes [37].

As we have already identified, risk-based notions of water resources management are well established [38], though their uptake in practice has been patchy [17], both in scope and scale. Flood risk management is now the prevailing paradigm for responding to floods [36,39]. In water resources, the scope of application has been rather narrow, for example focusing upon reservoir optimization [40–42]. Here, our aims are much more broad based, seeking to demonstrate how concepts of risk can underpin a multi-attribute definition of water security.

## 3. Formalizing the risk definition

We consider some appropriate spatial scale of an aquatic system. It need not be self-contained in aquatic terms: there will be fluxes over the boundary. The system will almost invariably have been modified, often profoundly so, by human influences. The system is defined in terms of a vector of state variables, **w**(*t*), where *t* denotes time dependency, which define the aquatic attributes of the system: storage volumes (above and below ground), water levels, flows, water quality indicators, soil moisture contents, precipitation and so on.

Drawing upon the conceptual framework proposed in the Millennium Ecosystem Assessment [43], we define a set of water-related services that the aquatic system performs, which are associated with constituents of human well-being. Aquatic systems are dealt with extensively in the Millennium Ecosystem Assessment and are recognized for their role in *provisioning* water for use domestically, in agriculture and industry, *regulating* floods and assimilating waste and *cultural* services, for example recreation.

The attributes **a** that are valued in the aquatic system are a function *g* of a subset of the system states (which may include antecedent conditions as well as, or instead of, the present state) and a series of time-dependent auxiliary variables, **x**(*t*), which determine the human and biophysical factors that go together with the aquatic system state variables to determine the ecosystem services that the aquatic system provides. Each attribute in the vector **a** is calculated as a function of the system state and auxiliary variables. *a*_*i*_(*t*) may be a function of past (time *t*−*n*) as well as present (time *t*) system states—it may, for example, be sensitive to cumulative effects. Thus,
3.1
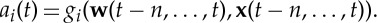

These attributes **a** that are valued in the aquatic system might include

— the frequency of water shortages to domestic or industrial water users,
— the frequency of violating water quality standards,
— counts of aquatic species,
— the output of irrigated agricultural land, and
— the distribution of flood damages.

We note that the relative influence of aquatic variables **w** and auxiliary variables **x** upon these outcomes varies. We also note that the function *g*_*i*_ can be applied to future (uncertain) system states, to estimate which outcome would be associated with a given vector of future conditions. Given the variation in time of **w** and **x**, the corresponding variation in **a** may be described by a joint probability density function *f*(**a**). We can therefore calculate expectations of *a*_*i*_ or probabilities of *a*_*i*_ exceeding given thresholds, for given time frames.

**Figure 3.**
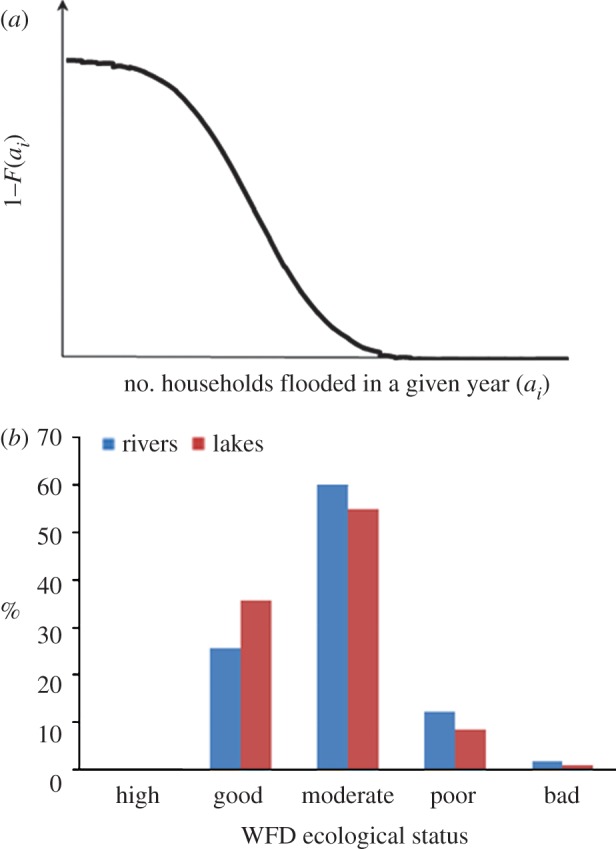
Examples of summary representations of water-related attributes for populations of actors. (*a*) Households flooded. (*b*) Ecological status of rivers and lakes in England. (Online version in colour.)

Attributes may be disaggregated with respect to different actors in the system: some residents may get flooded, others may not; some farmers may have water for irrigation, others may not. Particularly informative are frequency distributions of the numbers of actors who do, or do not, satisfy some target for a water-related attribute (figure 3).

**Figure 4.**
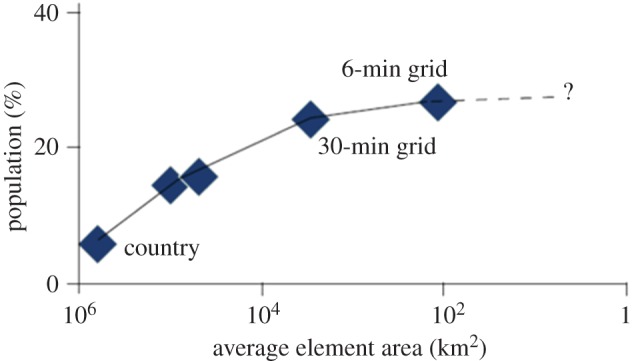
Water stress in Africa as percentage of the population, computed with increasing resolution [4]. (Online version in colour.)

Computing probability distributions of attributes across actors requires information about the dependence between outcomes for different actors. Large-scale events, for example major droughts, which impact upon multiple actors, are often highly correlated with space and time. The incidence of correlated risk is of particular interest from the perspective of national governments concerned about water security, or insurers who may be providing cover for water-related risks. Understanding spatial correlation is essential to understand how risks aggregate with spatial scale. Countrywide risk estimates may conceal smaller locations of high risk, in particular when populations are concentrated in areas of higher risk (figure 4). By the same token, tolerability of risk will depend on the size of population, with societies tending to be averse to very large-scale losses to people [44].

**Figure 5.**
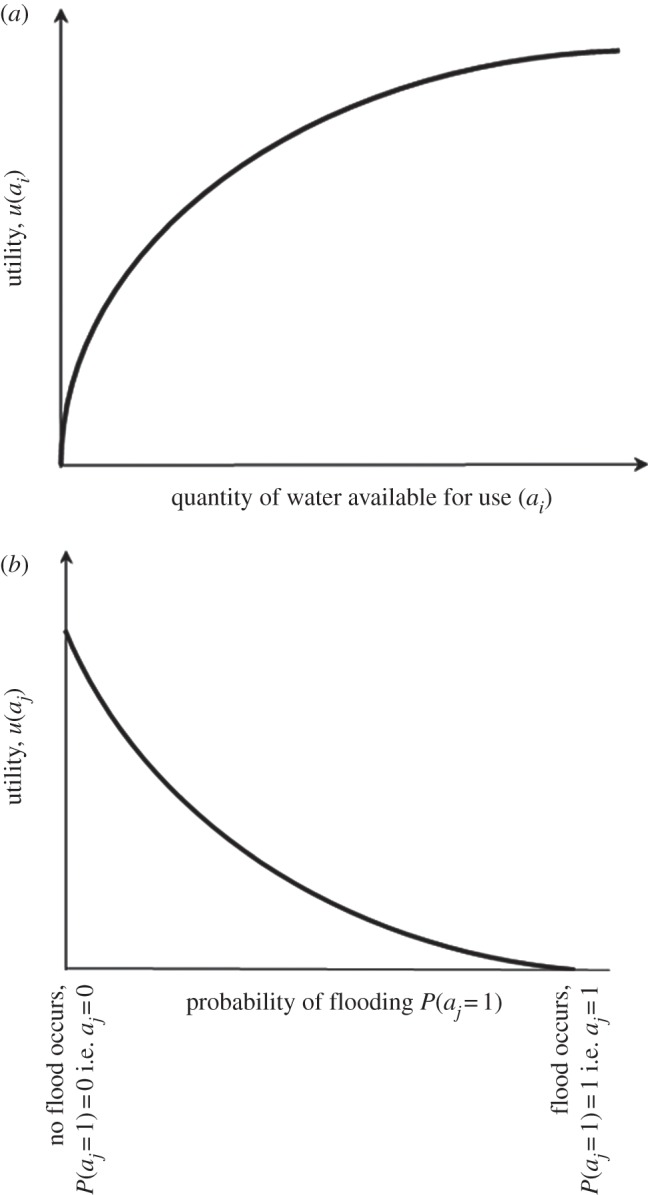
Utility representation of significant characteristics of water security. (*a*) Decreasing marginal value and (*b*) risk aversion.

Actors associate utilities with water-related attributes **a**. Their utilities will reflect both their preferences with respect to different combinations of attribute settings and their attitudes to risk. This is only usually practical if it is possible to obtain a representation of the utility function such that
3.2
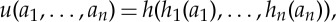

where *h*_*k*_ is a function of attribute *a*_*k*_ only and where *h* has a simple form, for example additive or multiplicative. Nonetheless, a functional form of this type does enable us to represent two important characteristics of water which lead to a risk-based framing of water security: decreasing marginal utility and risk aversion (figure 5). The significance of decreasing marginal utility is, as has been discussed above, rather central to our definition of water security in terms of tolerable risk, and has long been regarded as a defining (and, for Adam Smith, puzzling) characteristic of water [16]. Risk aversion, in particular in relation to low-probability–high-consequence events, helps to explain why governments continue to invest in costly hazard management schemes, even when the expected risk is relatively low [16].

Risk-based decision making involves choices between alternative courses of actions on the basis of their benefits in terms of risk reduction and their costs, aggregated through time over some appropriate time horizon. All of these costs and benefits are aggregated into the utility function in equation (3.2), which then provides the rationale for establishing a preference ordering over alternative courses of action.

Although complete and coherent as an approach to decision making, two significant reservations should be noted at this point. The first is the practical difficulty of eliciting utilities in practical settings [45]. In practice, the economic benefits of water services may be quite diffuse and embedded in many aspects of economic value, so are difficult to unravel [16]. Secondly, the preference ordering of options may be sensitive to uncertainties in our understanding of the system in question and the utilities associated with it. Several authors have highlighted the importance of exhaustive sensitivity testing in order to identify decisions that are as far as possible robust to uncertainties [37,46–48].

## 4. A framework for indicators of water security

**Figure 6.**
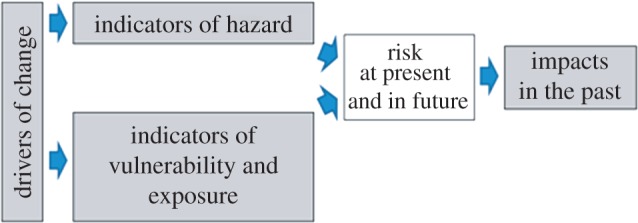
Components of risk-based indicators of water security. Observable quantities are highlighted. (Online version in colour.)

Risk is not an observable quantity, so if we base a definition of water security upon risk, it means that water security is not directly observable. This presents a problem if we wish to develop indicators that will track risk through time and demonstrate the effectiveness (or otherwise) of adaptations that are intended to reduce risk. A complete picture of risk can be constructed from a composite set of indicators that track the various factors that contribute to risk (figure 6). Indicators of hazard will include the frequency of hydrological extremes, harmful water quality and so on. They may extend to indicators of deliberate water security threats such as terrorist attack or dependence on hostile neighbouring nations. Indicators of exposure relate to the numbers of exposed populations, businesses, species, etc. Indicators of vulnerability relate to the sensitivity of species and the coping capacity of individuals and communities. A composite of these indicators can be used to track change through time and elucidate the factors that may influence or explain change.

While risk is associated with events that may, or may not, occur in the future, past sequences of events and their impacts provide some evidence about risks even though (i) conditions may have changed and (ii) the past record is a sample of possible outcomes, which may not be a reliable guide, in particular with respect to extreme events. Nonetheless, given appropriate contextualization, observations of impacts from harmful water-related events provide useful evidence of water security. Outcomes that are observed are realizations of a risk. These may include numbers of households affected by flooding, records of agricultural outputs, observations of decline in species population or diversity, and so on.

Given that risks are changing with time for a variety of reasons, it is also necessary to monitor indicators of change in relevant variables. These may be reported as rates of change, or they may be implicit in times series of indicators. Evidence of future changes may come from model studies. Particular insights may be gained from monitoring factors that are influencing rates of change in hazard and vulnerability, which are often referred to as the ‘drivers’ of change [49]. These include climatic, demographic or economic changes. Metrics of these changes, both in the past and future projections, provide further evidence with which to contextualize and attribute risks.

A composite indicator of risk would combine the indicators of hazard and consequence with some statistics of realized damage. However, more insight can be acquired by keeping these indicators separate, but retaining the structure illustrated in figure 6 as a means of relating these indicators to one another and understanding their relevance to risk. An example of indicators structured in this way is provided by the Adaptation Sub-Committee (ASC) of the UK's independent Committee on Climate Change. In its 2012 report [50], the ASC developed indicators of the risks of flooding and water scarcity. The ASC's focus was upon indicators of (i) exposure and vulnerability, (ii) realized losses (impacts), and (iii) adaptation actions. Indicators of hazard were given less attention in the indicator set because long-standing hydro-meteorological monitoring programmes already exist in the UK. The indicators of water-scarcity risk (table 1) demonstrate how security of supply has increased and over the long term the number of drought orders has decreased. However, demand is going up, both domestically and in agriculture. The UK's Climate Change Risk Assessment indicates that if these trends continue in the context of a changing climate, the risk of water shortage may become unacceptable [51].

**Table 1.** Summary of indicators to risk of water (England unless otherwise stated). The direction of the arrow represents the trend in the indicator (increasing, decreasing and no significant trend). The signs inside the arrows represent the implications of trends in terms of risk (plus sign, increasing risk; minus sign, decreasing risk; equal sign, risk is neither increasing nor decreasing). Adapted from the ASC [50].

indicator of	indicator name	source	long-term (10 yr +) observed trend	most recent year trend (2011 or 2012)	time series length
indicators of risk (exposure and vulnerability)					
supply	security of supply index (SOSI)	Ofwat	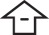	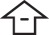	2002–2012
overall demand	freshwater abstraction (non-tidal) by sector	Environment Agency	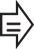	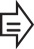	1995–2009
household demand	average *per capita* consumption—all households	Ofwat	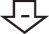	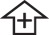	2000–2011
household demand	average *per capita* consumption—metered households	Ofwat	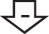	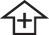	2000–2011
household demand	average *per capita* consumption—unmetered households	Ofwat	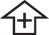	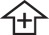	2000–2011
agricultural demand	average volume of water applied for irrigation per hectare by crop type	Ofwat	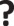	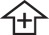	2005 and 2011
indicators of action					
reducing demand	% properties with water meters (England and Wales)	Ofwat	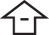	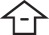	2000–2012
increasing supply	total leakage (England and Wales)	Ofwat	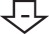	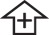	1992–2011
indicators of impact					
water availability (public water supply)	% of reservoir capacity filled (England and Wales)	Defra	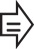	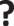	1988–2009
water availability (economic)	catchments where water is available for abstraction (England and Wales)	Environment Agency	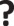	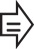	2009–2011
water availability (environmental)	compliance with Environmental Flow Indicators (England and Wales)	Environment Agency	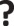	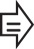	2009–2011
water availability (social)	number of drought orders	Defra	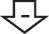	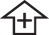	1976–2012
water availability (social)	number of water companies issuing hosepipe bans (England and Wales)	Environment Agency	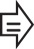	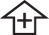	1974–2012

Construction of a risk-based indicator set, even in a relatively data-rich setting like England, has not been straightforward. Nor does the ASC's indictor set yet represent an ideal set of metrics of exposure, vulnerability and impact. However, it does demonstrate how a composite indicator, based on principles of risk, can be constructed and used to illustrate the evolution of risk and the effectiveness of adaptation actions.

## 5. A simulation framework for water security assessment

Indicators provide a mechanism for monitoring evolving risk through time. However, risk-based decisions require predictions of the ways in which risks may change in future. These predictions inevitably require the use of models. Water resource systems models, coupled increasingly with economic assessment tools [52], are a mainstay of water planning and appraisal decision making. Yet, the deployment of these models in support of project appraisal and decision making has tended not to be explicitly risk-based, nor to lend itself to the assessment of adaptive management *strategies*, as opposed to fixed management actions.

For example, in England the conventional approach to water resources planning compares conservative estimates of water availability and demand, and seeks to optimize a portfolio of actions that maintain a margin between supply and demand. The approach is not suited for incorporating probabilistic treatment of a range of uncertainties, in particular climate uncertainties [48]. The arrangements are not explicitly risk based, so it is impossible to determine whether planned management actions are in proportion to the risks. While the margin between supply and demand (‘headroom’) can be regarded as a metric of water security, it does not provide information about the likelihood of observable events of interest to water users (water shortages of different degrees of severity) occurring.

A risk-based approach to appraisal relies on the ability to predict (in the context of attendant uncertainties) the probability and consequences of observable outcomes of different degrees of severity. Testing alternative strategies for adapting to risks requires a simulation framework which can track possible sequences of events and decisions through time. Here, we propose such a framework with a focus upon risks of water scarcity for domestic, industrial and agricultural users. The approach is based upon the testing of large numbers of synthetic hydrological sequences, which enables exploration of hydrological variability. By resampling different parameter settings in the model, it is possible to explore the implications of a variety of different sources of uncertainty, including uncertainties in future climate, catchment response and future demands for water.

**Figure 7.**
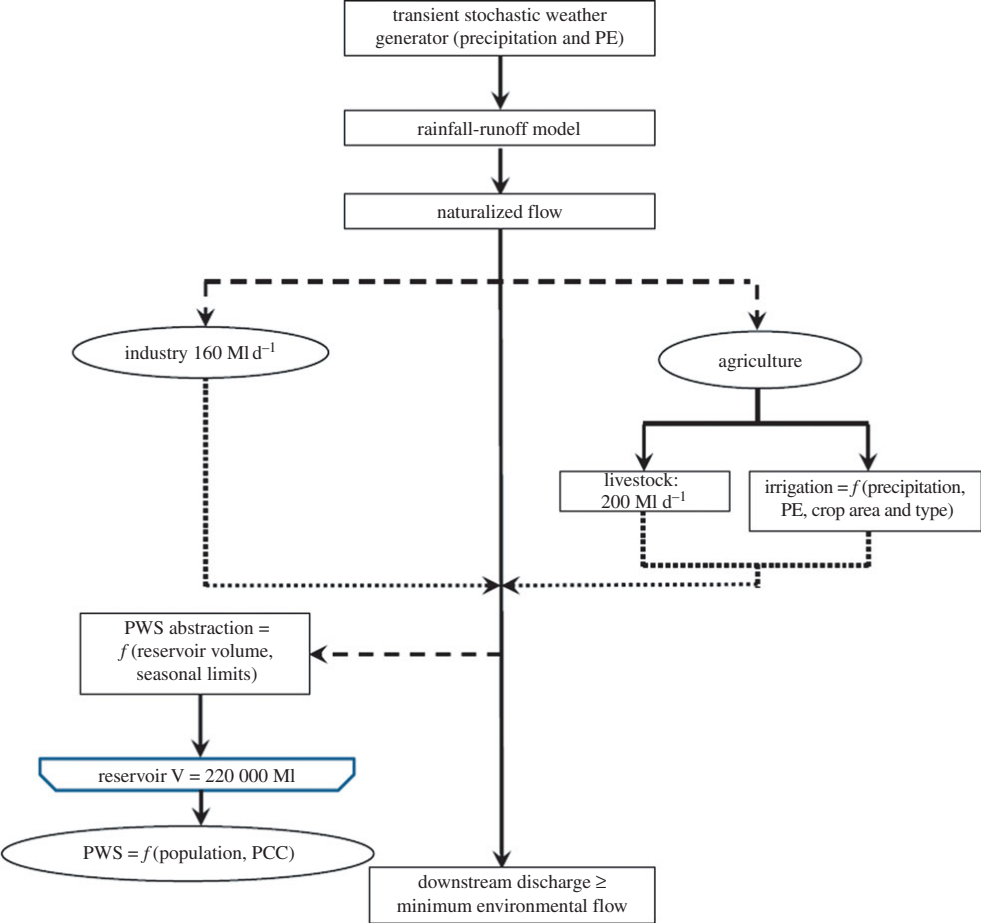
Overview of modelling framework. PE, potential evapo-transpiration; PWS, public water supply; PCC, *per capita* consumption. Dashed lines represent river abstractions and dotted lines represent return flows. (Online version in colour.)

The system simulation for a typical river basin in England is summarized in figure 7. The upstream input is provided by stochastic sequences of rainfall, temperature and potential evapo-transpiration (calculated using the Penman–Monteith equation), propagated through a conceptual rainfall-runoff model [53]. The weather generator preserves autocorrelation at a monthly scale but has no autocorrelation at an interannual scale. This is consistent with UK weather statistics which show no significant interannual autocorrelation. Methodology is under development to explore the sensitivity to interannual drought persistence.

**Figure 8.**
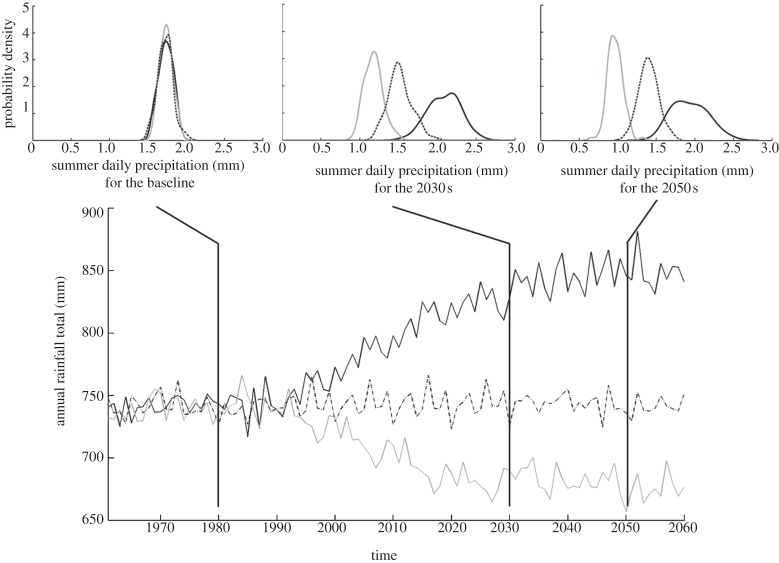
Typical synthetic time series of total annual precipitation for three different sets of climate change factors. Insets illustrate the evolution of probability density functions through time for summer mean catchment daily precipitation.

The possible range of future climatic conditions is obtained from the large perturbed physics ensemble of climate model simulations undertaken for the UKCP09 climate scenarios [54,55]. This two-layered approach to probabilistic uncertainty analysis incorporates natural variability (via the stochastic weather generator) and epistemic uncertainty in future climate (by randomly resampling the climate change factors from the perturbed physics ensemble) [56]. Figure 8 shows three different synthetic sequences, which start by being sampled from the same baseline climatology but then diverge, because each one is conditioned on a different set of climate change factors. The insets in figure 8 illustrate how the probability distributions of precipitation corresponding to these three sets of change factors (obtained empirically by repeated realization of the stochastic process) evolve through time.

Surface flows for the system are allocated using an algorithm that partitions available water between four different uses: environmental, domestic, industrial and agricultural. Environmental requirements are applied as a constraint on abstractions, which vary according to the time of year. Domestic, industrial and agricultural demands are based upon a range of possible demand factors for population scenarios and *per capita* demand (domestic) and agricultural production. In the baseline case, 83% of water abstractions are used for public water supply, while 11% are used for agriculture and 6% for industry, representing a typical situation in southern England. Crop water requirements are computed using the crop coefficient approach [57,58]. While most crop area is used for cereals, they only account for 10% of water abstractions for irrigation. Potatoes consume 47% of the irrigated water volume and other vegetables a further 30%. Thus, a shift to a greater proportion of land used for horticulture would have major implications on water demand.

Water may be used directly or may be stored in the case of the public water supply (domestic use). At times of scarcity (as signified by low reservoir levels), various measures to reduce demand may be introduced, with corresponding effects in terms of demand reduction, as specified in table 2.

**Table 2.** Demand reduction measures and their effect on domestic demand.

restriction level	target frequency of occurrence	water use restrictions	expected demand reduction (cumulative) (%) [59]
level 1	1 year in 5 on average	intensive media campaign	2.2
level 2	1 year in 10 on average	sprinkler/unattended hosepipe ban, enhanced media campaign	9.1
level 3	1 year in 20 on average	temporary use ban (formerly hosepipe ban)	13.3
level 4	‘never’	standpipes and rota cuts, request of an emergency drought order	31.3

**Figure 9.**
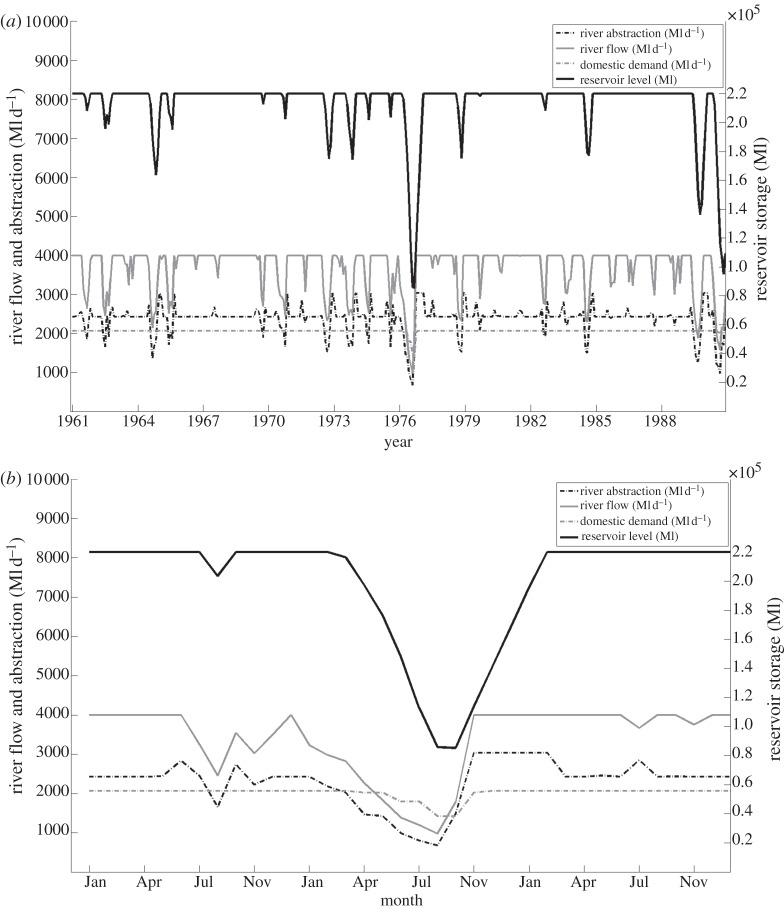
River flows, abstractions and reservoir levels (for public water supply) for observed rainfall: (*a*) 1961–1990; (*b*) 1975–1977. Flows are capped at 4000 Ml d^−1^ for clarity.

Figure 9 illustrates the time series of abstractions and reservoir levels for the baseline period of 1961–1990, which includes the major drought of 1976, which was the only occasion during the baseline period where water restrictions had a notable impact on demand. River abstractions were restricted in order to preserve environmental flows on more frequent occasions.

The simulation framework provides the capacity to track the frequency of potentially harmful events, namely (i) water shortages of different severity for different users and (ii) environmentally harmful low flows. The consequences of these harmful events are not explicit, though the extent of vulnerable populations, industries and habitats is known. However, a risk-based metric of tolerability is implicit in the target levels of service set out in table 2. These are based upon willingness to pay surveys of customer preferences [60].

**Figure 10.**
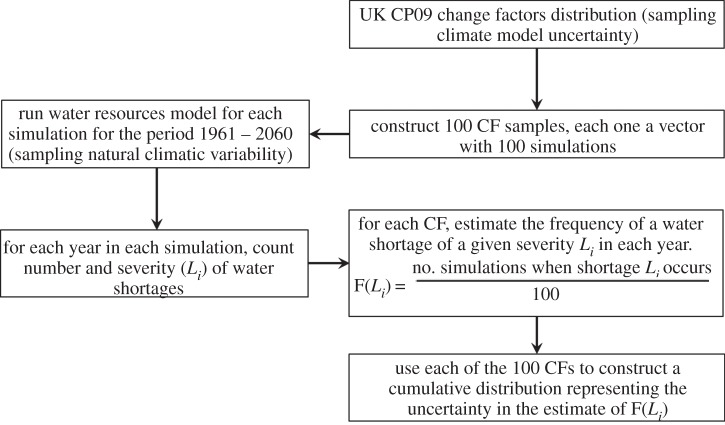
Overview of simulation routine for calculation of probabilities of meeting tolerable frequencies for water shortages.

**Figure 11.**
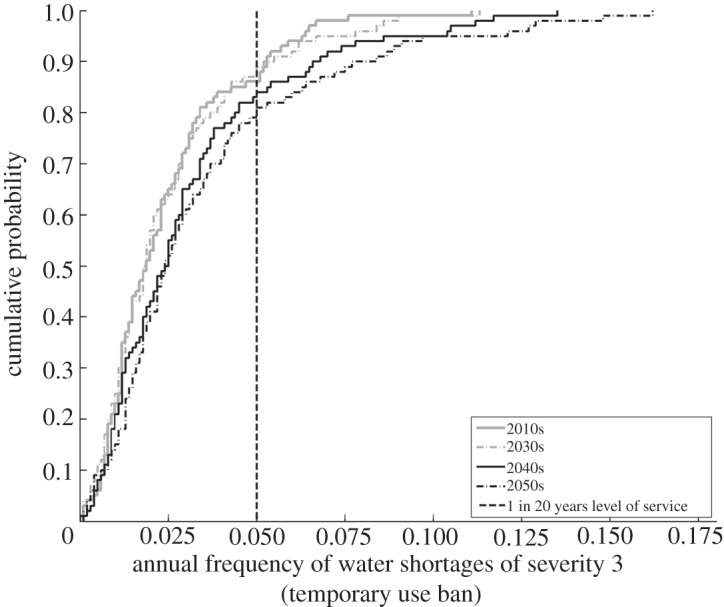
Simulated frequency of shortages of given severity. Vertical dashed line indicates the target level of service.

For simulations of the future, repeated stochastic realizations based upon the same assumptions will yield a frequency of water shortages of different levels of severity (figure 10). This can be compared with the target frequency (the vertical dashed line in figure 11) in order to calculate the probability of failing to meet the target (at the point where the cumulative curve crosses the target line), which is the risk-based indicator for domestic water scarcity to domestic water users.

**Figure 12.**
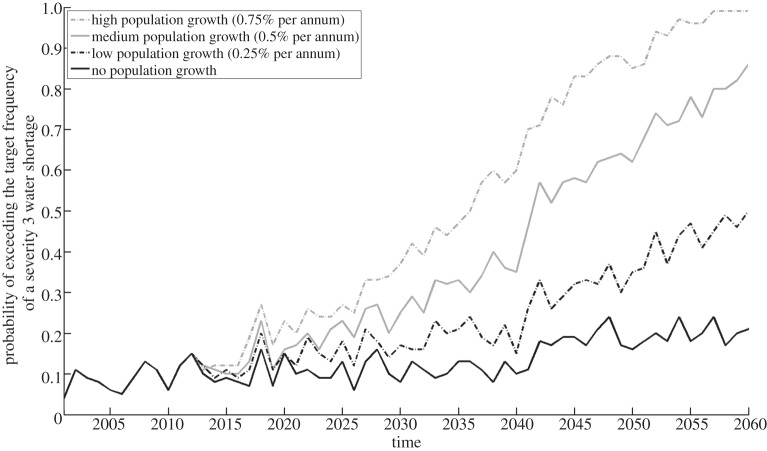
Evolution of the probability of failure to meet level of service 3 (a temporary use ban once in 20 years on average), for different rates of population growth, assuming no change in supply or *per capita* demand.

Figure 12 illustrates how the probability of failure to meet the target frequency (once in 20 years) for temporary use bans evolves, given no modifications on the supply side, for different rates of population growth and a medium emissions scenario. While the system is only moderately sensitive to low and medium rates of population growth, it becomes very sensitive to high rates of population growth. This occurs when the steep part of the cumulative distribution of shortages shifts to be near the target for tolerable risk (figure 11), so the system becomes very sensitive to changes on the demand side.

**Figure 13.**
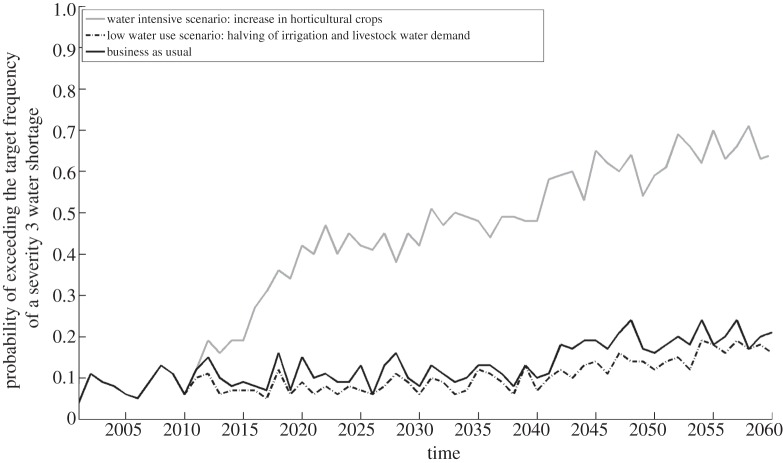
Evolution of the probability of failure to meet level of service 3 (a temporary use ban once in 20 years on average), for given changing agricultural demand, assuming no change in supply or *per capita* demand.

Figure 13 illustrates how plausible reductions or increases in upstream agricultural abstractions could have an impact upon risks to domestic customers. These scenarios represent rather major changes in agricultural demand. Demand reductions will not have an appreciable effect on the risk to domestic water supply. A scenario in which there is a significant shift to horticultural production is not implausible if domestic production were to have to substitute for imports of horticultural products from locations that could in future be adversely affected by a changing climate. This would however have a notable effect on the water balance in the basin and the risks to other water users.

While the study described above is idealized in many respects and only captures the roles of a small number of actors in the water resources system, it does illustrate how analysis of a variety of sources of uncertainty and variability can contribute to understanding of how water-related risks will evolve through time. In particular, we have observed how risks for different users are traded off against one another and how future uncertainties, including highly uncertain non-stationary climatic conditions, can be incorporated within this risk-based framework.

The simulation study has focused upon the generation of probabilistic indicators of the future severity of water shortage. In other words, the work has focused upon indicators of hazard (figure 6). Metrics of exposure are incorporated in terms of the populations and industries that are at risk, which are projected to change at uncertain rates through time. Metrics of vulnerability are implicit in the targets set for frequencies of water shortages and minimum environmental flows. Further progress towards risk-based decision making would require a more explicit quantification of these impacts.

## 6. Risk in complex, uncertain and contested settings

Our framing of water security in terms of risk reflects an increasing emphasis on risk-based approaches in policy making and governance. Risk is attractive because it provides a way of incorporating scientific knowledge and some of the associated uncertainties, articulating it in a way that provides a normative route to decision making. More broadly, risk practices fit with current advanced liberal forms of government, with their emphasis upon economic rationality [61]. They appeal to the notion that risks are controllable by risk-management actions.

However, this convenient route to ‘science-based’ decisions has been criticized when knowledge is uncertain and contested. In particular, scholars including Keynes [62] and Knight [63] have argued that probabilistic representation of uncertainty may not be justifiable in the context of severe uncertainty. Adopting a probabilistic representation of uncertainty when it is not warranted by the available evidence can lead to assessments of risk that underestimate the total uncertainty and adoption of management responses that are vulnerable to those uncertainties. In response, Stirling [64] promotes plurality of approaches, to explore alternative versions of uncertainty. Scholars have advocated approaches that can incorporate ambiguity in available evidence [65] and imprecision in estimates of probabilities [66]. There is an increasing emphasis on analysis of the robustness of decisions to severe uncertainties [22,67].

Alongside the difficulties of quantifying uncertainties when evidence is scarce or conflicting, risk-based approaches are also prone to difficulties of valuation, in particular in the context of long-term changes which require discounting, incommensurable values and situations where costs and benefits are unequally distributed. The multi-attribute utility theory we have advocated provides a mechanism for articulating these various values, and attitudes to their associated uncertainties, as utilities. Yet, we acknowledge in particular that elicitation of preferences for environmental goods and services is problematic conceptually and methodologically [61] and in practice can yield widely ranging estimates. The difficulties of reaching collectively acceptable decisions when there are multiple actors with different utility functions are fundamental [68].

Thus, great care is required in using the evidence from analysis of risks to inform public policy decisions. At worst, risk analysis can be a mechanism for excluding legitimate perspectives, in particular on uncertainty, and coercively reaching particular management outcomes. On the other hand, we argue that transparently implemented risk analysis provides a mechanism for exposing the implications of uncertainty for outcomes that people value. It provides a structure for integrating multiple perspectives and objectives with respect to water resources systems in a way that, at its best, provides a platform for deliberative decision processes and expert critique.

## 7. Conclusion

Explanations for the chronic issues of water insecurity worldwide are diverse, but tend to have their roots in a lack of proper appreciation by water users in an increasingly crowded world of the multiple values that water yields, and of hydrological variability and change on a range of different time scales. Given the increasing human demands placed upon water resources systems and human vulnerability to water-related hazards, it is clear that continuation of current practices will only make matters worse. One of the few sources of optimism is in the increasing availability of information to understand the behaviour of water resources systems and explore possible system responses to scenarios on a range of time scales and from the perspectives of multiple actors. This information has the potential to transform management practices and provide a platform for inclusive decision making.

However, scientific insight into the processes of change in water resources systems alone is insufficient to lead to sustainable management practices. That information needs to be orientated towards the sets of management options in order to inform understanding of their potential impact in terms of outcomes that people value. Here, we have argued that it is *harmful* outcomes that are of most concern. We have observed that water management in practice seems to be designed to manage the frequency of undesirable water-related outcomes, be they related to shortage or excess of water, inadequate access or harmful water quality. In other words, water management focuses upon the management of water-related risks. In a transition to water security, societies seek to reduce risks to a level at which they are broadly tolerable, when compared with other risks in society and in the context of the costs of further risk reduction. We therefore endorse Grey *et al*.'s [6] notion of water security being a tolerable level of water-related risks. Definition of water security in terms of tolerable risk is intuitively appealing. It is also operational, in that it provides a direct connection with decision making that seeks cost-effective ways of reducing risks to tolerable levels.

While acutely aware of the limitations of probabilistic methods for dealing with severe uncertainties, we are attracted by risk-based methods because of their incorporation of the natural variability that is characteristic of hydrological systems. Superimposed upon that natural variability is a range of epistemic uncertainties related to system functions now and potential changes in the future. In an example of a water resources system, we have demonstrated how these two levels of uncertainty can be incorporated in water resources assessment. The example also illustrated the trade-offs in risks among different water users. Moreover, we have demonstrated that while risk is not an observable quantity, the structure of a risk calculation, and the recognition that past harmful impacts are realizations of risk, provides the basis for development of indicators of water security.

Because our definition of risk embraces hazards, vulnerability and exposure, it means that risk can be used to distinguish between a very wide range of possible options for water management, including interventions in both biophysical and human systems. More specifically, risk provides a direct route to comparing and choosing between a range of possible water management options and deciding upon the appropriate scale of implementation of different options. Comparison of risks and costs helps to identify whether the costs, in the broadest sense, of proposed management actions are in proportion to the risks. We do not suppose that risk provides a uniquely uncontested route to decision making. Risk-based decisions should be open to scrutiny and tested for robustness from a variety of different perspectives. However, risk does provide a rational framework for structuring available evidence and exploring costs, benefits and trade-offs.
